# Proteasomal Inhibition Redirects the PrP-Like Shadoo Protein to the Nucleus

**DOI:** 10.1007/s12035-019-1623-1

**Published:** 2019-05-25

**Authors:** Sang-Gyun Kang, Charles E. Mays, Nathalie Daude, Jing Yang, Satyabrata Kar, David Westaway

**Affiliations:** 1 Centre for Prions and Protein Folding Diseases, University of Alberta, 204 Brain and Aging Research Building, Edmonton, Alberta T6G 2M8 Canada; 2 Division of Neurology, University of Alberta, Edmonton, Alberta Canada; 3 Department of Biochemistry, University of Alberta, Edmonton, Alberta Canada

**Keywords:** Nuclear localization, Proteasomal inhibition, Proteostasis, Impaired ER import, Neurons and glia

## Abstract

The Shadoo protein (Sho) exhibits homology to the hydrophobic region of the cellular isoform of prion protein (PrP^C^). As prion-infected brains gradually accumulate infectivity-associated isoforms of prion protein (PrP^Sc^), levels of mature endogenous Sho become reduced. To study the regulatory effect of the proteostatic network on Sho expression, we investigated the action of lactacystin, MG132, NH_4_Cl, and 3-methyladenine (3-MA) in two cell culture models. In primary mixed neuronal and glial cell cultures (MNGCs) from transgenic mice expressing wild-type Sho from the PrP gene promoter (Tg.*Sprn* mice), lactacystin- and MG132-mediated inhibition of proteasomal activity shifted the repertoire of Sho species towards unglycosylated forms appearing in the nuclei; conversely, the autophagic modulators NH_4_Cl and 3-MA did not affect Sho or PrP^C^ glycosylation patterns. Mouse N2a neuroblastoma cells expressing Sho under control of a housekeeping gene promoter treated with MG132 or lactacystin also showed increased nuclear localization of unglycosylated Sho. As two proteasomal inhibitors tested in two cell paradigms caused redirection of Sho to nuclei at the expense of processing through the secretory pathway, our findings define a balanced shift in subcellular localization that thereby differs from the decreases in net Sho species seen in prion-infected brains. Our data are indicative of a physiological pathway to access Sho functions in the nucleus under conditions of impaired proteasomal activity. We also infer that these conditions would comprise a context wherein Sho’s N-terminal nucleic acid–binding RGG repeat region is brought into play.

**Electronic supplementary material:**

The online version of this article (10.1007/s12035-019-1623-1) contains supplementary material, which is available to authorized users.

## Introduction

Prion infections result in a conformational remodeling of the cellular isoform of the prion protein called PrP^C^, yielding a beta-sheet enriched and infectivity-associated isoform denoted PrP^Sc^ [1]. In support of this concept, subtle mutations in the prion protein gene (*Prnp*) in mice impact disease progress and pathology [2–4]. PrP^C^ knockout mice (*Prnp*^0/0^) have normal development and are completely resistant to prion infections [5]. Given PrP^C^’s conservation (catalogued by Wopfner et al. [6]), the viability of homozygous null mice was in some regards surprising [7] and provoked the hypothesis of a cryptic functional homolog [8]. Subsequent to these speculations, the Shadoo protein (Sho) gene (*Sprn*) was found by its homology to the PrP^C^’s central hydrophobic region [9]. Further work established fish and mammalian Sho are glycoproteins synthesized in the secretory pathway and attached to the external face of the plasma membrane via a glycosylphosphatidylinositol (GPI) anchor [10, 11]. Sho is expressed in the mouse central nervous system (CNS) in regions that partially overlap with PrP^C^ expression and the two proteins share a number of binding partners in common. Despite these similarities, double knockout *Sprn*^0/0^ + *Prnp*^0/0^ mice are viable [12, 13], suggesting that Sho is not a functional homolog of PrP^C^ or that other homologs exist in the mouse genome.

Refolding and aggregation are touchstones in PrP biology and, notably, natively disordered recombinant Sho (amino acids 25–122) has a propensity to aggregate to a protease-resistant form in vitro [14, 15]. Protease resistance of full-length Sho also has been reported in mammalian cells under certain conditions interfering with proteostasis [16]. In prion infections where proteinase K–resistant isoforms of PrP^Sc^ (resPrP^Sc^) are accumulating and where protein degradation mechanisms have an extra burden, the levels of mature Sho do not increase. Rather, they decrease [11, 17–19], with a similar effect being deduced for PrP^C^ [20]. These parallel findings raise the broader question of the concerted proteostatic systems relevant to the reduction of both Sho and PrP^C^. However, the work in this area encompasses diverse and sometimes conflicting findings.

Causal roles of the altered Sho regulation following prion infection have been attributed to glycosylation, in mediating stable expression, and the proteasomal pathway in degrading unglycosylated Sho [16, 21]. Alternative subcellular targeting may be an important variable in this mechanism. There is extensive literature concerning a leaky signal peptide for PrP^C^ that does not always engage the endoplasmic reticulum (ER) translocon [22–25]. Sho is reported to be directed to mitochondria under conditions of an impaired ER import [26] and Sho-YFP fusion proteins have been reported in the nucleus in a number of different cell lines under resting conditions [27]; this latter situation could arise from the use of nuclear targeting signals that apply to proteins synthesized from cytoplasmic ribosomes. Untagged wild-type Sho has been demonstrated in the nucleus as well as in membrane fractions in sequential purifications of mouse brain samples [13].

To tackle these diverse results and the question of proteostasis for the mammalian prion protein family, we have used experimental systems that include primary mixed neuronal and glial cell cultures (MNGC’s) and immortalized neuroblastoma cells expressing Sho (N2a-*Sprn*). Our studies reveal glycosylation state as an indicator (rather than a determinant) of cellular decision to induce nuclear localization of Sho under conditions of proteasomal inhibition. These data align with the nucleic acid binding and subcellular targeting properties of Sho’s RGG repeat region [27–29] and offer a basis for understanding divergent aspects of Sho and PrP^C^ biology defined by genetic studies [13, 30, 31].

## Materials and Methods

### Cell Cultures: Mixed Neuronal and Glial Cell Culture, and N2a-*Sprn*

Primary mixed neuronal and glial cell cultures (MNGCs) were prepared from both transgenic mice expressing full-length Shadoo protein (Sho) (Tg.*Sprn*^+/–^) and Sho knockout mice (*Sprn*^0/0^) [30] as described previously [32]. All animal handling protocols were in accordance with Canadian Council on Animal Care and University of Alberta institutional ethics review (protocol AUP00000356). One day before the culture, genomic DNA of the transgenic mice was extracted from toe snips and genotyped for the presence or absence of the *Sprn* transgene by polymerase chain reaction (PCR) using the following primer pairs. 3′UTR forward: 5′-TCGATCCAGAGCCTTTGAATTGAG-3′ and 3′UTR reverse: 5′-GGGTGAAATGGTCAGTGCATTACG-3′. The next day, MNGCs were extracted from cerebella of 8-day-old pups (Tg.*Sprn*^+/–^ and *Sprn*^0/0^) by mechanical and enzymatic dissociation with trypsin. The cells were plated on plastic culture dishes coated with 10 μg/mL poly-l-lysine (PLL, Sigma-Aldrich, MO, USA, P4707) at 1 × 10^6^ cells/well in a six-well plate. The cells were maintained at 37 °C with 5% CO_2_ in minimum essential medium (MEM, Sigma-Aldrich, M4655), supplemented with 10% fetal bovine serum (FBS, Gibco, 12438), 25 mM KCl, and penicillin-streptomycin (Gibco, MA, USA, 15140). For serum starvation, the cells were not supplemented with FBS for 16 h.

Murine neuroblastoma cells, N2a (ATCC, VA, USA, CCL-131), and chronically prion-infected N2a [33, 34] were maintained at 37 °C with 5% CO_2_ in Dulbecco’s modified Eagle’s medium (DMEM) with high glucose (4.5 g/L) and 2 mM glutamine (Gibco, 11995-065), supplemented with 10% FBS and penicillin-streptomycin. The coding region for full-length wild-type Sho (*Sprn*) was cloned into a mammalian expression vector, pBudCE4.1 (Invitrogen, CA, USA, V532-20), in which Sho expression was driven either by a human elongation factor 1 alpha (EF-1α) promoter or by a cytomegalovirus (CMV) promoter. N2a cells were transfected with the *Sprn* constructs using Lipofectamine 2000 reagent (Invitrogen, 11668) and stable cell clones were obtained by zeocin (Invitrogen, R250) selection. To determine Sho expression, the transfectants were harvested and lysed in RIPA lysis buffer (1% Triton X-100, 1% sodium deoxycholate, 150 mM NaCl, 50 mM Tris-HCl, pH 7.4, 0.1% SDS, 1 mM EDTA) containing a protease inhibitor cocktail (Roche, 04 693 159 001).

### Modulation of the Intracellular Proteolytic System

MNGCs (at 7 days in vitro culture) and N2a-*Sprn* (at second passage) were treated with modulators targeting proteolytic system, with conditions specified as per figure legends. These modulators included lactacystin (Sigma-Aldrich, L6785), MG132 (Selleck Chemicals, TX, USA, S2619), NH_4_Cl (Sigma-Aldrich, 213330), and 3-methyladenine (3-MA, Sigma-Aldrich, M9281), and innate immune ligands including lipopolysaccharide (LPS, Sigma-Aldrich, L5668) and polyinosinic:polycytidylic acid (poly I:C, Sigma-Aldrich, P1530). The cells were then harvested with RIPA lysis buffer containing a protease inhibitor cocktail.

### Western Blot

The protein concentration of cell lysates was measured by BCA protein assay (Pierce, MA, USA, 23235). To detect unglycosylated forms of Sho and cellular isoform of the prion protein (PrP^C^), N-linked glycans were removed by PNGase F treatment (25 units/μL, New England Biolabs, MA, USA, P0704) at 37 °C for 1 h. The samples were resolved on 15% Tris-Glycine gels or NuPAGE Bis-Tris gels (Invitrogen, NP0343) and transferred to PVDF membrane (Thermo Fisher Scientific, MA, USA, 88518). The membrane was blocked with 2% bovine serum albumin (BSA, Darmstadt, Germany, 2960) in TBST (TBS with 0.1% Tween 20) and probed with monoclonal (mAb) or polyclonal (pAb) antibodies at 4 °C overnight: anti-Sho pAb, 06SH1 [11]; anti-PrP mAb, Sha31 (Spibio, France, A03213); anti-ubiquitin pAb (Santa Cruz, TX, USA, sc-9133); anti-lysosomal-associated membrane protein 1 (Lamp-1) pAb (Abcam, Cambridge, UK, ab24170); anti-microtubule-associated protein 1A/1B-light chain 3 (LC3) pAb (MBL, Nagoya, Japan, PM036); anti-glyceraldehyde 3-phosphate dehydrogenase (GAPDH) mAb (Abcam, ab9484); anti-histone H3 pAb (Abcam, ab1791); anti-heat shock protein 60 (Hsp60) pAb (Abcam, ab46798); anti-β-actin mAb (Abcam, ab20272). Anti-mouse or anti-rabbit IgG antibodies conjugated to horseradish peroxidase (Bio-Rad, CA, USA, 170-6516 and 170-6515) or alkaline phosphatase (Promega, MI, USA, S327B or S323B) were used as the secondary antibodies and visualized by detecting chemiluminescence (Pierce, 32209) or fluorescence (Promega, S1000) signals. The membrane was stripped in western blot stripping buffer (Thermo Fisher Scientific, 46430) and re-probed as needed.

### Immunocytochemistry

Cells were plated on PLL-coated microscope cover glass (Thermo Fisher Scientific, 12-545-83) or SlideFlasks (Nunc, NY, USA, 170920). For immunocytochemistry, cells were fixed in paraformaldehyde (4%, pH 7.4) for 15 min and optionally permeabilized with PBS containing Triton X-100 (0.1%). The fixed cells were blocked with 1% BSA in PBST (PBS with 0.1% Tween 20) for 30 min and probed with mAb or pAb at 4 °C overnight: anti-Sho pAb, 06SH1 and 06SH3 [11]; anti-PrP mAb, SAF83 (Cayman, MI, USA, 189765); anti-microtubule-associated protein 2 (MAP2) pAb (Abcam, ab5392). To visualize the target molecules, cells were then incubated with appropriate fluorescent-conjugated secondary antibodies (Alexa Fluor 488 or Alexa Fluor 594, Invitrogen). Counterstaining for nuclei was performed with Hoechst 33342 (Invitrogen, H1399). Cells were then imaged by a confocal microscopy (LSM700 laser scanning microscope, Zeiss, Jena, Germany) with Z-stack functions under identical imaging settings. The images were analyzed by Zen 2010b SP1 imaging software (Zeiss) and ImageJ (https://imagej.nih.gov/ij/).

### Subcellular Fractionation

To determine the translocation of target molecules, nuclear and cytoplasmic extracts were prepared using NE-PER Nuclear and Cytoplasmic Extraction Reagents (Thermo Fisher Scientific, 78833) following the manufacturer’s instructions. Cells were harvested after trypsinization for cell counting. Then, input materials were normalized by pelleting 2 × 10^6^ cells from each treatment. Cell membranes were disrupted by addition of the first detergent. Cytoplasmic extracts were recovered by centrifugation and the nuclei were then lysed with a second detergent to yield nuclear extracts. The purities of the extracts were determined by western blot probed with anti-GAPDH mAb (Abcam, ab9484) and anti-histone H3 pAb (Abcam, ab1791).

### Statistical Analysis

The number of independent experiments or biological replicates of compared groups was at least *n* = 2 for each observation. Statistical analysis for western blot results was performed using the independent sample *t* test and one-way analysis of variance (ANOVA) followed by post hoc Tukey’s honest significant difference (HSD) test. Statistical analysis of all data was performed using PRISM (GraphPad Software, CA, USA) version 5 software.

## Results

### Proteasomal Inhibition and Sho Expression in MNGCs

**Fig. 1:**
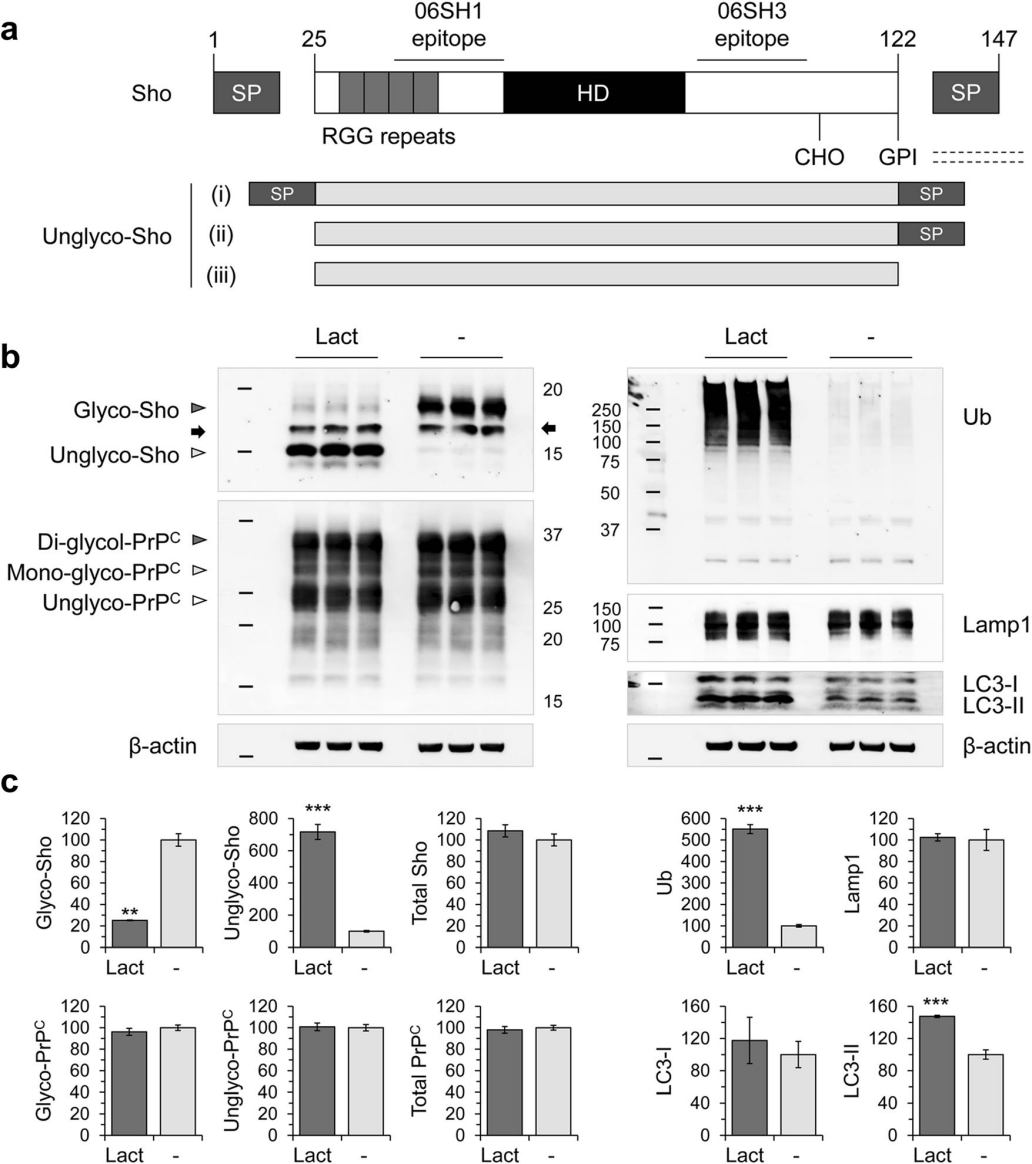
Effect of lactacystin on Sho and PrP^C^ expression in mouse MNGCs. **a** Domain structure of the Shadoo protein (Sho). N-terminal region to the hydrophobic domain (HD) includes tandem positively charged RGG boxes (25–61). A single N-linked glycosylation site is located in the C-terminal region (CHO, residue 107) (top). Anti-Sho pAb, 06SH1 and 06SH3 have N-terminal epitope (30–60) and C-terminal epitope (86–100), respectively. In western blot analysis, Sho appears as a 22-kDa glycosylated band (mature form), or 10–15-kDa unglycosylated bands depending on cleavage of the signal peptides (bottom). (i) unprocessed with both signal peptides intact; (ii) N-terminal signal peptide removed; (iii) both N- and C-terminal signal peptides removed. **b** Primary MNGCs were derived from Tg.*Sprn* mice and treated with lactacystin at 1 μM concentration for 16 h (*n* = 3). Expression of Sho, PrP^C^, ubiquitin (Ub), lysosome-associated membrane protein 1 (Lamp1), and microtubule-associated protein light chain 3 (LC3-I and LC3-II) was analyzed by western blot. A cross-reactive band is indicated with an arrow. Arrowheads indicate glycosylation of Sho (Glyco, glycosylated; Unglyco, unglycosylated) and PrP^C^ (Di-glyco, di-glycosylated; Mono-glyco, mono-glycosylated; Unglyco, unglycosylated). In the absence of PNGaseF treatment, PrP^C^ fragments as well as full-length protein retain intact glycosylation sites, contributing to a complex electrophoretic profile). Molecular masses based on the migration of protein standards are shown in kDa. **c** Intensity measurement of western blot results in **b**. Intensities were normalized to those of β-actin loading controls. Error bars represent SD. **p < 0.01 and ***p < 0.001 in comparison with vehicle (water) treatment controls

Sho and the cellular isoform of prion protein (PrP^C^) differ in their configuration of N-terminal natively disordered sequences—Sho contains RGG repeats, while PrP^C^ contains PHGGGWGQ metal-binding octarepeats (Fig. 1a) [9, 11]. Downregulation of glycosylated forms of Sho is a preclinical molecular signature of prion diseases [11, 18–20]; however, the cellular mechanism remains unclear. To test the more general question as to whether inhibition of proteolytic systems causes Sho downregulation, four modulators targeting the proteostatic network were tested in MNGCs from Tg.*Sprn* (Tg.*Sprn*-MNGC) and *Sprn*^0/0^ knockout mice (*Sprn*^0/0^-MNGC): (1) lactacystin which modifies the 20S proteasome subunit β5 and irreversibly blocks its activity [35], (2) MG132 that reduces degradation of ubiquitinated proteins through the 26S proteasome by interfering with catalytic activity [36], (3) NH_4_Cl which attenuates lysosomal acidification, causing a reduction in lysosome-mediated protein degradation and disruption of autophagolysosome formation [37] and (4) 3-methyladenine (3-MA) that inhibits type III phosphoinositide 3-kinase (PI-3K) and thereby attenuates formation of phagophores [38].

**Fig. 2:**
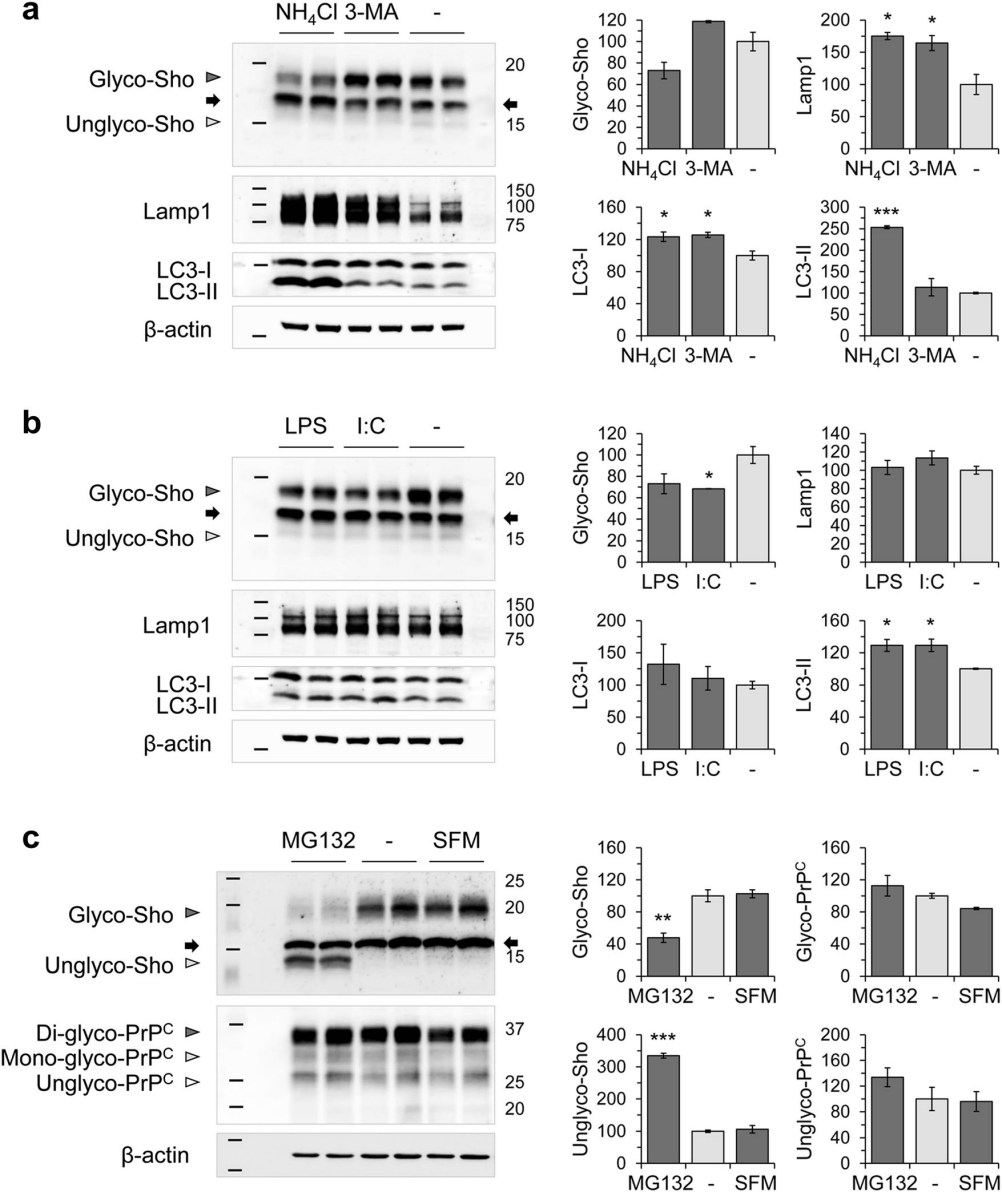
Effects of autophagic modulators and innate immune ligands on Sho expression in MNGCs. Primary MNGCs were derived from Tg.*Sprn* mice and treated with **a** autophagic modulators (NH_4_Cl or 3-MA at 10 mM concentration) or **b** innate immune ligands (LPS or poly I:C at 10 μg/mL and 100 μg/mL concentration, respectively) for 16 h (*n* = 2). Expression of Sho, lysosome-associated membrane protein 1 (Lamp1), and microtubule-associated protein light chain 3 (LC3-I and LC3-II) was analyzed by western blot. **c** To verify an alteration in glycosylation profiles, which is mediated by MG132 (at 1 μM) rather than a transient stress condition, Tg.*Sprn*-MNGCs were maintained in serum-free medium (SFM) for 16 h (*n* = 2). Expression of Sho and PrP^C^ was analyzed by western blot. A cross-reactive band is indicated with an arrow as per Fig. 1. Intensity measurements of western blot results are presented. Intensities were normalized to those of β-actin loading controls. Error bars represent SD. *p < 0.05, **p < 0.01, and ***p < 0.001 in comparison with vehicle (water or dimethyl sulfoxide (DMSO)) treatment controls

Proteasomal inhibition by a non-peptide inhibitor, lactacystin, increased polyubiquitinated protein levels (550.8 ± 20.8%) in Tg.*Sprn*-MNGCs compared with vehicle treatment controls (as 100%). Protein levels of lysosome-associated membrane protein 1 (Lamp1) and microtubule-associated protein light chain 3 (LC3-I) were unaltered, while LC3-II increased (147.4 ± 1.4%), as previously reported [39, 40] (Fig. 1b, c). Lactacystin-mediated proteasomal inhibition decreased glycosylated forms of Sho (25.1 ± 0.4%) and increased unglycosylated forms (716.4 ± 46.9%), thereby not changing total levels of Sho, which were estimated as the sum of glycosylated and unglycosylated forms. PrP^C^, although belonging to the same protein superfamily as Sho, did not demonstrate a change in glycosylation profile (Fig. 1b, c). Autophagy-lysosome pathway modulations by NH_4_Cl and 3-MA significantly increased levels of Lamp1 (175.3 ± 5.7% with NH_4_Cl and 164.4 ± 11.7% with 3-MA) and LC3-I (123.5 ± 6.0% with NH_4_Cl and 125.8 ± 3.4% with 3-MA). While lysosomal inhibition with NH_4_Cl caused an increased LC3-II levels (253.5 ± 2.9%) compared with vehicle (water) treatment. These treatments, however, did not significantly affect total levels and glycosylation profiles of Sho in Tg.*Sprn*-MNGCs (Fig. 2a). Proteasomal function can be compromised by the neuroinflammation which often accompanies neurodegeneration [32, 41–44], and as Sho has a nucleic acid–binding region [29] suggestive of a role in molecular pattern recognition after infections, we also tested the effects of two innate immune ligands. Although glycosylated Sho levels were mildly decreased by these treatments (*p* = 0.0295 for poly I:C), unlike the effect of proteasomal inhibitors, we were unable to detect a commensurate increase in level for the unglycosylated forms of Sho (Fig. 2b). The proteasomal inhibition effect was further confirmed using a peptide aldehyde inhibitor, MG132. Like lactacystin, MG132 altered glycosylation of Sho (glycosylated Sho, 47.8 ± 5.9%; unglycosylated Sho, 334.7 ± 7.5%) without changing total Sho levels, while MNGCs maintained in serum-free medium (SFM)showed no alteration in glycosylation profile of both Sho and PrP^C^ versus non-treated controls (Fig. 2c). These data indicate that proteasomal inhibition interfered with ER import of Sho and caused an abundance of unglycosylated Sho isoforms with a corresponding decrease in levels of glycosylated Sho.

**Fig. 3:**
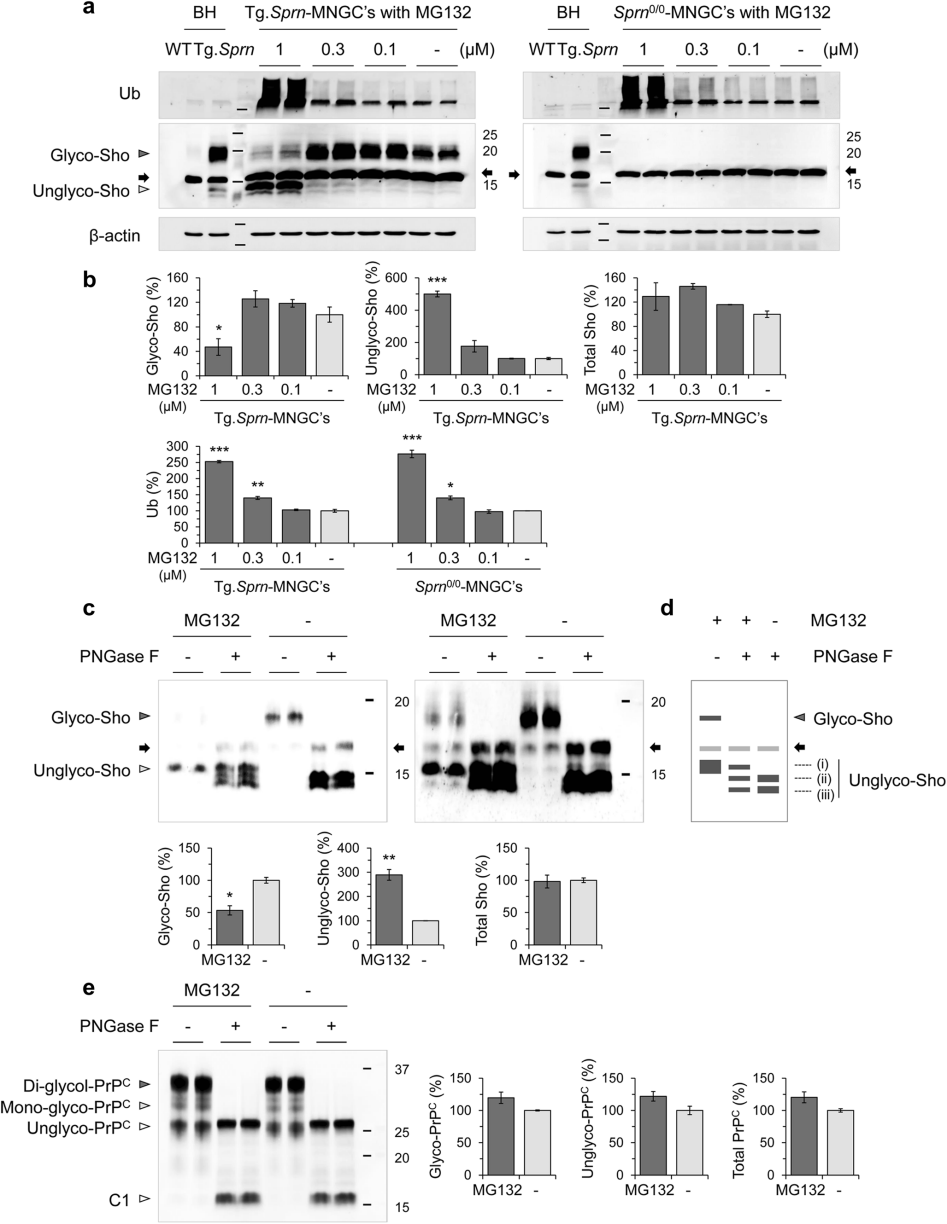
Dose-dependent effect of MG132 on Sho and PrP^C^ expression in MNGCs. **a** Primary MNGCs were derived from Tg.*Sprn* (left) and *Sprn*^0/0^ (right) mice, respectively. The cells were treated with MG132 at the indicated concentrations for 16 h (*n* = 2). Expression of Sho and ubiquitin (Ub) was analyzed by western blot with designations of glycosylated species and a cross-reactive band as per Fig. 1. Brain homogenates of wild-type (BH-WT) and Tg.*Sprn* (BH-Tg.*Sprn*) mice were loaded as positive controls. **b** Intensity measurement of western blot results in **a**. Intensities were normalized to those of β-actin loading controls. **c** Primary MNGCs derived from Tg.*Sprn* mice were treated with MG132 (1 μM) (*n* = 2). The cell lysates (10 μg) were incubated with PNGase F (25 units/sample) to digest N-linked glycosylation and then expression of Sho was analyzed by western blot. The top and bottom panels are identical except for the exposure time; note extra band at ~ 15-kDa (three-band versus two-band signature for PNGase F–treated samples) with MG132 treatment. Intensity measurements of the Sho bands are presented. **d** Diagram representing the electrophoretic mobility of Sho species observed in western blot results (**c**) with designations of protein species as per Fig. 1a. **e** PrP^C^ expression was analyzed in the same cell lysates tested in **c**. C1 designates a metabolically stable proteolytic product of PrP^C^ processing. Intensities of the PrP^C^ bands were measured. In the cell lysates without PNGase F, the relative amount of glycosylated proteins, including di-glycosylated and mono-glycosylated forms, and unglycosylated proteins is presented as a percentage to those of vehicle (DMSO) treatment controls. Error bars represent SD. *p < 0.05, **p < 0.01, and ***p < 0.001 in comparison with the vehicle controls

The dose-dependent effects of MG132 on Sho expression were examined in Tg.*Sprn*-MNGCs and *Sprn*^0/0^-MNGCs (which serve as a negative control culture). MG132 at 1 μM concentration increased polyubiquitinated protein levels in the both cultures, regardless of Sho transgene (252.6 ± 3.6% in Tg.*Sprn*-MNGCs and 276.4 ± 11.6% in *Sprn*^0/0^-MNGCs) (Fig. 3a, b). MG132-mediated proteasomal inhibition decreased glycosylated forms of Sho (47.2 ± 13.6%), whereas unglycosylated forms of Sho were increased (499.5 ± 18.1%) compared with vehicle treatment controls. The total Sho level including both glycosylated and unglycosylated forms was however unchanged (Fig. 3a, b). The increase in putatively unglycosylated forms implies an impaired Sho import into the ER. Nascent proteins destined to become GPI-linked have N- and C-terminal signal peptides cleaved off as they are processed through the secretory pathway (see Fig. 1a). To check the presence of processed forms of Sho in Tg.*Sprn*-MNGCs with MG132, we removed N-linked sugars with PNGase F to obviate confounding effects of sugar chains on electrophoretic mobility (Fig. 3c, d). In control cells with no MG132, two bands denoted as (ii) and (iii) as per Fig. 1a were observed after PNGase F treatment. Band (iii) corresponds to fully processed Sho while band (ii) is inferred to represent retention of the C-terminal signal peptide. PNGase F–treated samples from cells cultured with MG132 had an additional species, (i), inferred to represent retention of both signal peptides. This ~ 15-kDa species migrated in a similar but non-identical fashion to the species seen in MG132-treated cell lysates without PNGase F digestion (Fig. 3c, d) and it also had a similar signal intensity. These data suggest that the predominant form of Sho under conditions of proteasomal inhibition is unglycosylated and contains uncleaved N- and C-terminal signal peptides. In parallel experiments, glycosylation of PrP^C^ was not affected by the same MG132 treatment (Fig. 3e), indicating no effect of this drug on ER maturation.

### Nuclear Localization of Sho in MNGCs

**Fig. 4:**
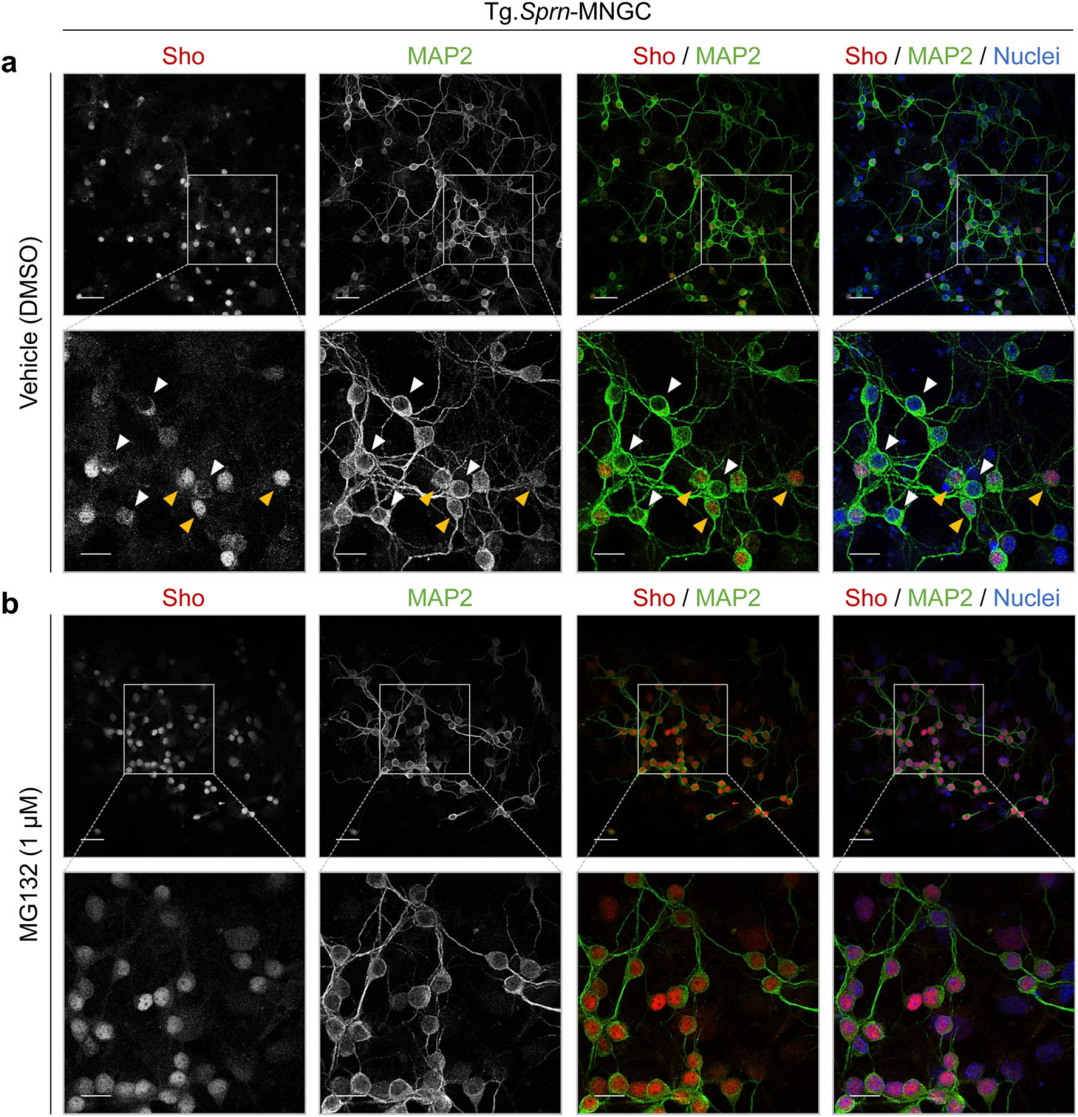
Nuclear localization of Sho in MNGCs with MG132 treatment. Primary MNGCs were derived from Tg.*Sprn* mice at postnatal day 7, then cultured for 7 days with the last 16 h of growth in the presence of MG132 (1 μM). The cells were then fixed and permeabilized. Sho (06SH1) and microtubule-associated protein 2 (MAP2) were probed with fluorescent-conjugated secondary antibodies. Sho in red; MAP2 in green. Nuclei were counterstained with Hoechst 33342 dye (blue). **a** Vehicle (DMSO) treatment controls showed intracellular localization of Sho signals (white arrowheads) with occasional diffuse nuclear staining in other cells (yellow arrowheads). **b** MG132 treatment triggered an increased nuclear localization of Sho. Scale bar, 20 μm and 10 μm in the boxed images

**Fig. 5:**
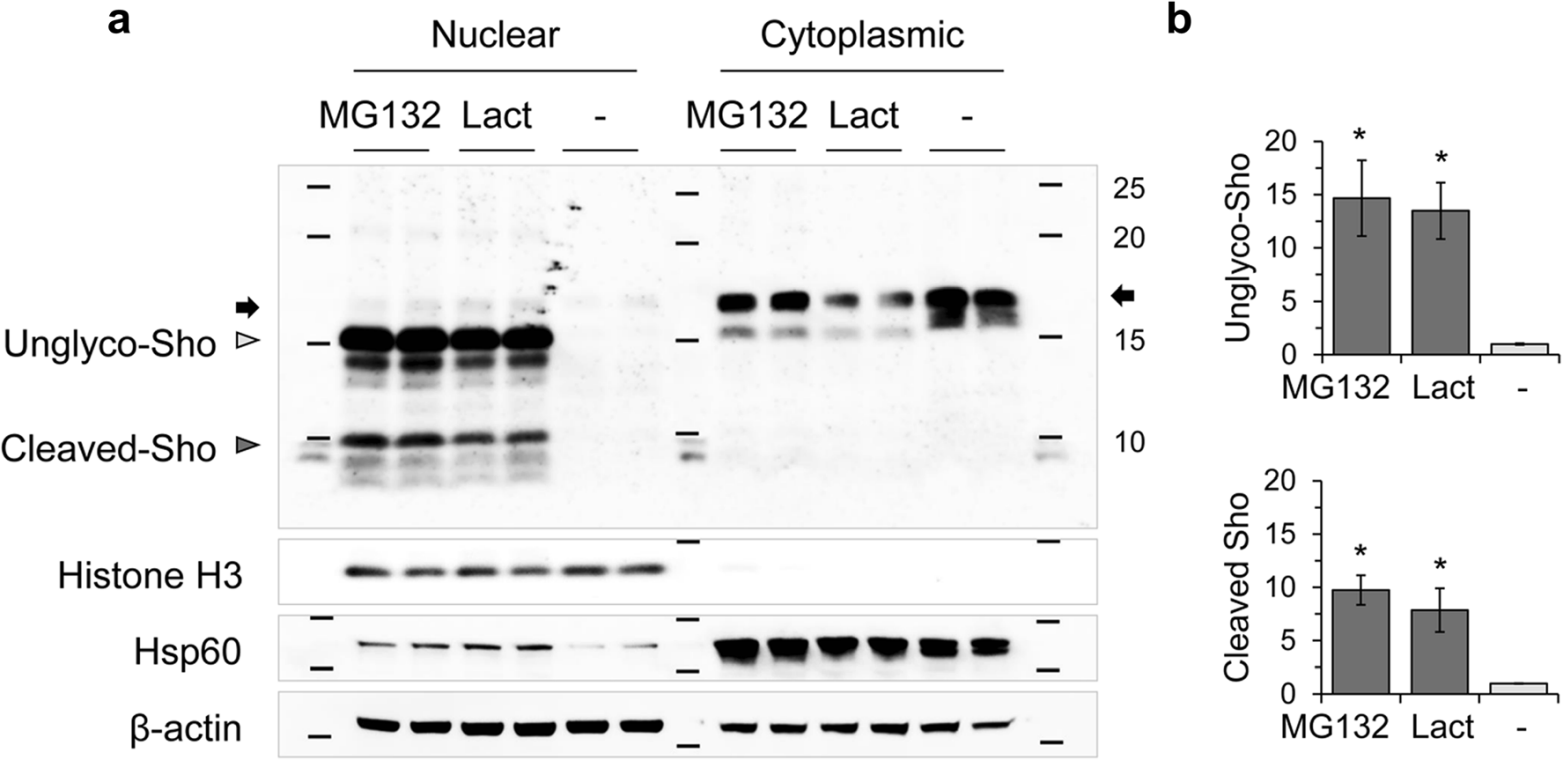
Subcellular fractionation of MNGCs treated with MG132 and lactacystin. **a** Tg.*Sprn*-MNGCs were treated with MG132 (1 μM) or lactacystin (Lact, 1 μM) for 16 h and harvested after trypsinization (*n* = 2). Nuclear and cytoplasmic extracts were prepared by differential detergent fractionation. Nuclear localization of Sho under proteasomal inhibition conditions was analyzed by western blot of the subcellular fractions. Histone H3, a nuclear marker; Hsp60, heat shock protein 60, a mitochondrial marker; β-actin, a loading control. Note strong accumulation of a 15-kDa species (migrating faster than the cross-reactive band) putatively representing full-length unglycosylated Sho retaining both signal peptides. Signals indicative of candidate cleavage products were also prominently represented (versus being undetectable in matching cytoplasmic fractions). **b** Intensity measurement of nuclear Sho in western blot results (**a**). Intensities were normalized to those of histone H3. Error bars represent SD. *p < 0.05 in comparison with vehicle (DMSO) treatment controls. Designations of glycosylated species and a cross-reactive band are as per Fig. 1

MNGCs derived from Tg.*Sprn* mice were treated with MG132 and fixed for immunocytochemical analysis. As per earlier studies, intracellular signals were seen for wild-type Sho [11] and were observed in some microtubule-associated protein 2 (MAP2)–positive neurons with no treatment (vehicle controls), while other neurons had weak nuclear staining (Fig. 4a); it is fully possible that these nuclear signals represent cross-reactivity with an antigen other than Sho. In contrast, however, MG132 treatment (1 μM) increased the strength and uniformity of nuclear Sho signals in MAP2-positive neurons (Fig. 4b). To further verify the nuclear localization of Sho, nuclear and cytoplasmic fractions of Tg.*Sprn*-MNGCs were extracted by differential detergent fractionation and analyzed by western blot. The cells for this fractionation scheme were harvested after a trypsinization step, which would be anticipated to degrade glycosylated cell surface forms of Sho. Indeed, glycosylated Sho was barely visible in the cytoplasmic fractions. The ~ 15-kDa bands, corresponding to unglycosylated forms of Sho, were dominant in the nuclear fractions with both MG132 and lactacystin treatments (Fig. 5a, b). Interestingly, cleaved Sho bands smaller than 10 kDa in size were also detected in the MG132- and lactacystin-treated nuclear fractions of primary granule neurons and glial cultures (Fig. 5a).

### Proteasomal Inhibition and Sho Expression in N2a-*Sprn*

**Fig. 6:**
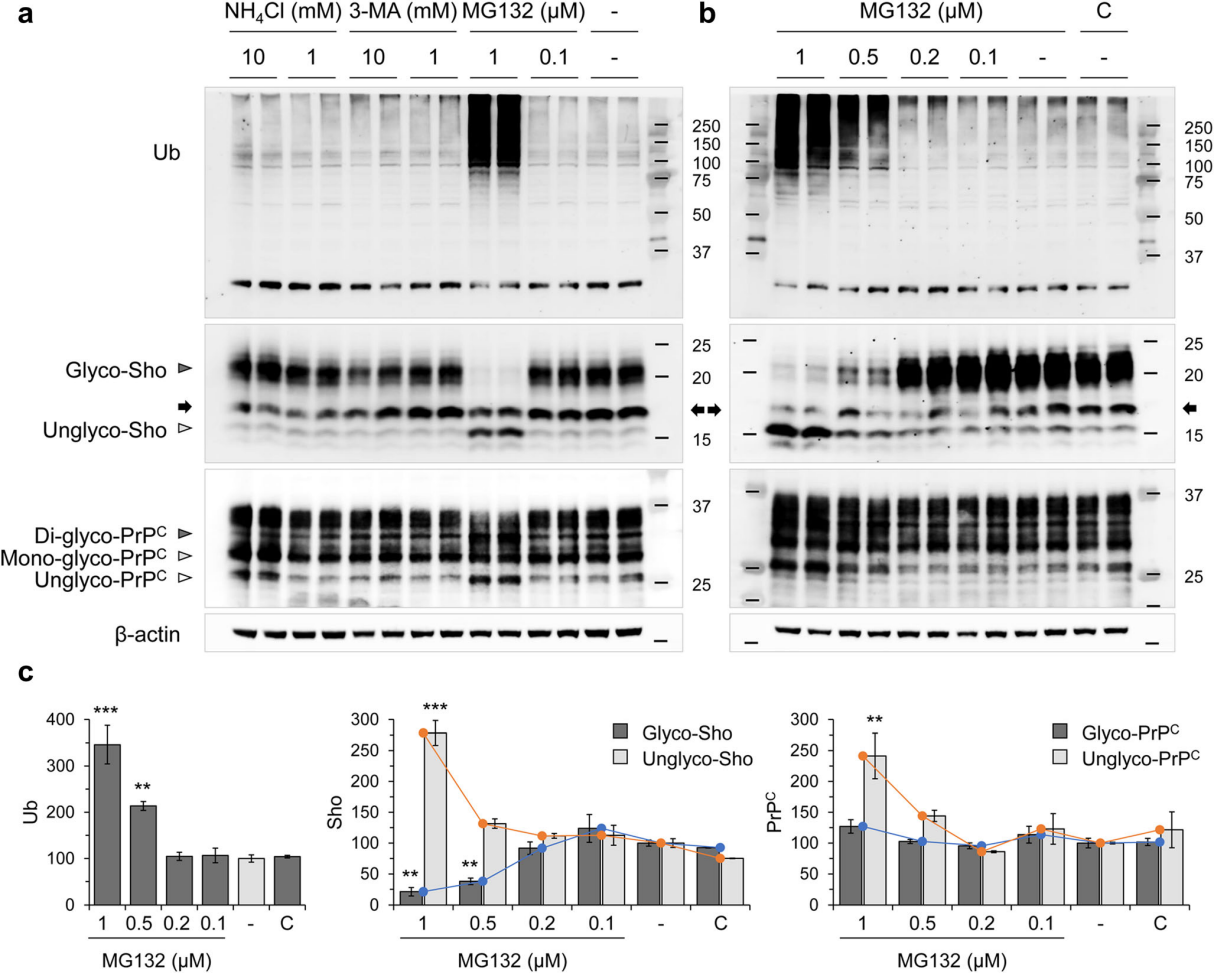
Selective effects of proteostatic modulators on Sho expression in N2a-*Sprn*. **a** N2a-*Sprn* cells were treated with the modulators targeting proteolytic system including MG132, NH_4_Cl, and 3-MA at the indicated concentrations for 16 h (*n* = 2). Expression of Sho, PrP^C^, and ubiquitin (Ub) was analyzed by western blot, revealing the effect of MG132 treatment versus lack of effects for NH_4_Cl and 3-MA. **b** Dose-dependent effect of MG132 on Sho and PrP^C^ expression in N2a-*Sprn*. The cells were treated with MG132 at the indicated concentrations for 16 h (*n* = 2). Expression of Sho, PrP^C^, and Ub was analyzed by western blot. C designates a normal culture condition (with no MG132 and no vehicle) while designations of glycosylated species and a cross-reactive band are as per Fig. 1. **c** Intensity measurement of western blot results in **b**. Intensities were normalized to those of β-actin loading controls. Error bars represent SD. **p < 0.01 and ***p < 0.001 in comparison with vehicle (DMSO) treatment controls

The effect of proteasomal inhibition on Sho expression was present in N2a murine neuroblastoma cells expressing Sho under control of human elongation factor 1 alpha (EF-1α) promoter (N2a-*Sprn*). N2a-*Sprn* cells were treated with the proteostatic modulators, including MG132, NH_4_Cl, and 3-MA, and expression of Sho species was again determined by western blot analysis. Like in Tg.*Sprn*-MNGCs, MG132 (1 μM) increased polyubiquitinated protein levels and caused glycosylation variation with a predominance of unglycosylated Sho over glycosylated forms (Fig. 6a). Investigation of the dose-dependent effect of MG132 in N2a-*Sprn* revealed that unglycosylated Sho increased with MG132, while glycosylated Sho showed an inverse relationship with increasing amounts of MG132 (Fig. 6b, c). Unglycosylated PrP^C^ increased with MG132 in a manner somewhat similar to Sho; however, glycosylated forms were little affected by the treatment doses (Fig. 6c).

### Nuclear Localization of Sho in N2a-*Sprn*

**Fig. 7:**
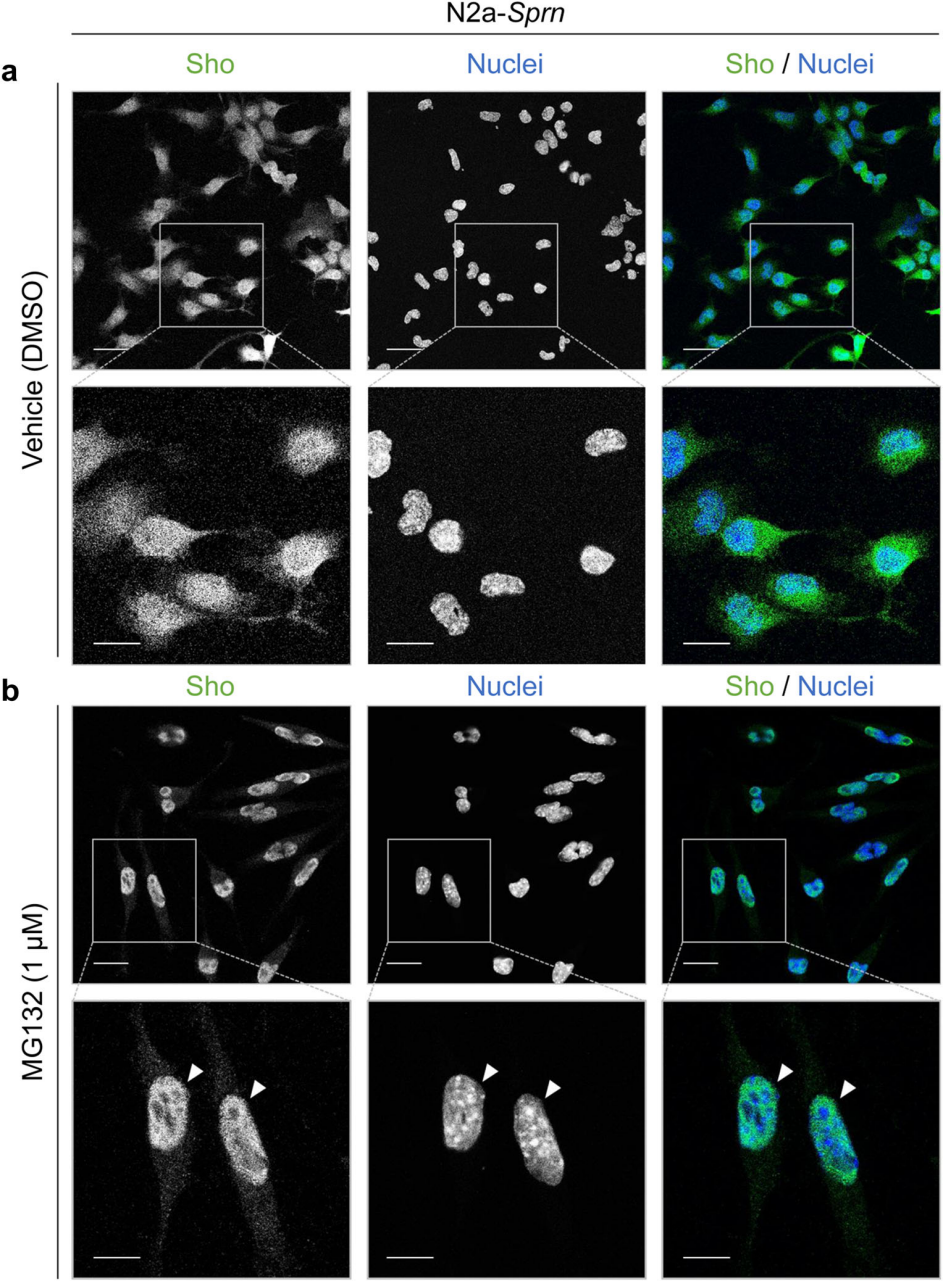
Nuclear localization of Sho in N2a-*Sprn* with MG132 treatment. N2a-*Sprn* cells were treated with MG132 (1 μM) for 16 h. The cells were then fixed and permeabilized. Sho was probed anti-Sho pAb, 06SH3. **a** Vehicle (DMSO) treatment controls showed a uniform distribution of Sho signals through the cell body. **b** MG132 treatment triggered a nuclear localization of Sho (white arrowheads). Sho in green. Nuclei were counterstained with Hoechst 33342 dye (blue). Scale bar, 20 μm and 10 μm in the boxed images

**Fig. 8:**
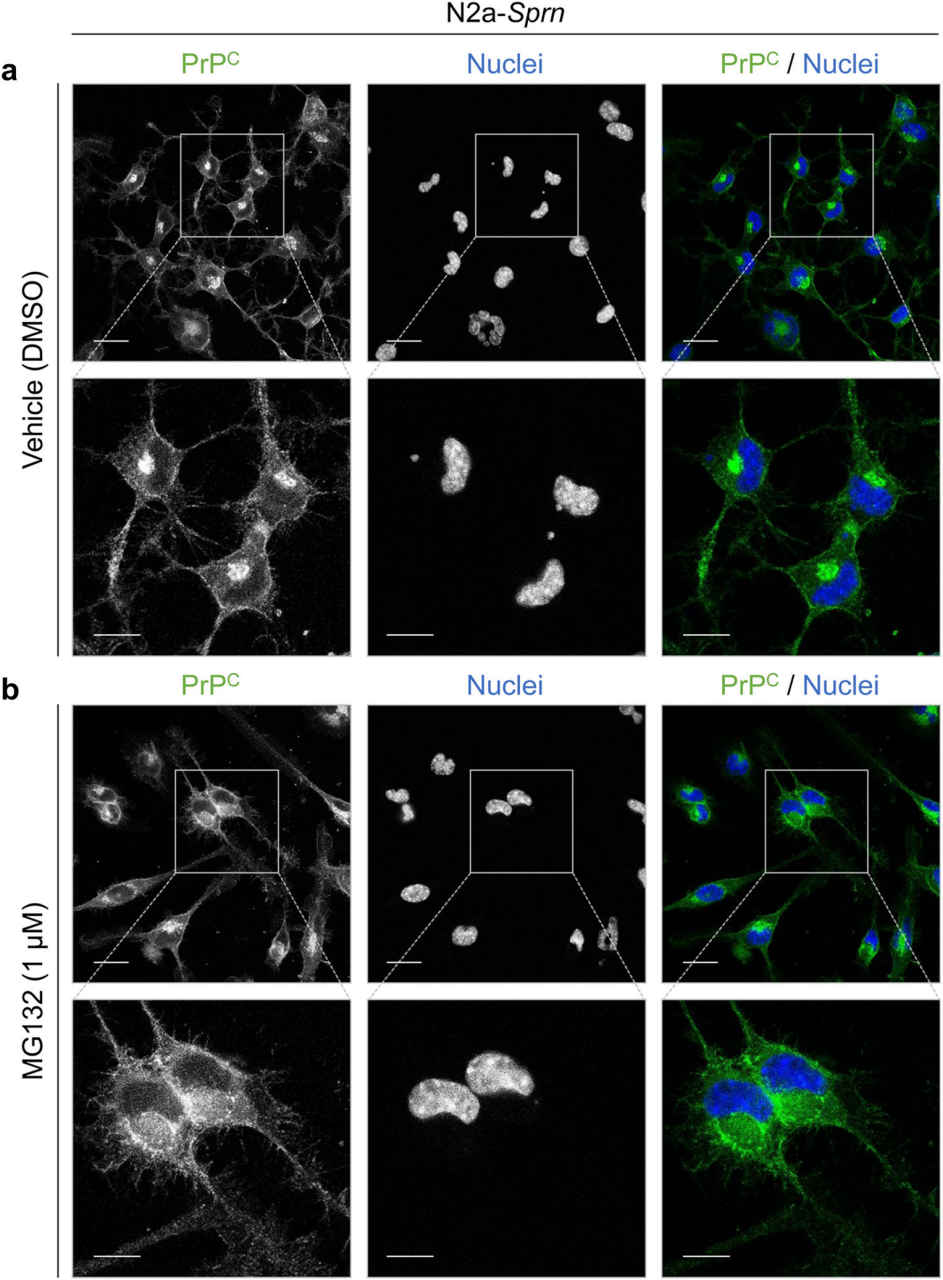
Cell surface and intracellular expression of PrP^C^ in N2a-*Sprn*. N2a-*Sprn* cells were treated with MG132. The cells were then fixed and permeabilized. PrP^C^ was probed with anti-PrP mAb, SAF83. Immunocytochemistry revealed cell surface and intracellular expression of endogenous PrP^C^ in N2a-*Sprn*, regardless of MG132 treatment. **a** Vehicle (DMSO) treatment control cells; **b** cells treated with MG132 treatment at 1 μM. PrP^C^ in green. Nuclei were counterstained with Hoechst 33342 dye (blue). Scale bar, 20 μm and 10 μm in the boxed images

**Fig. 9:**
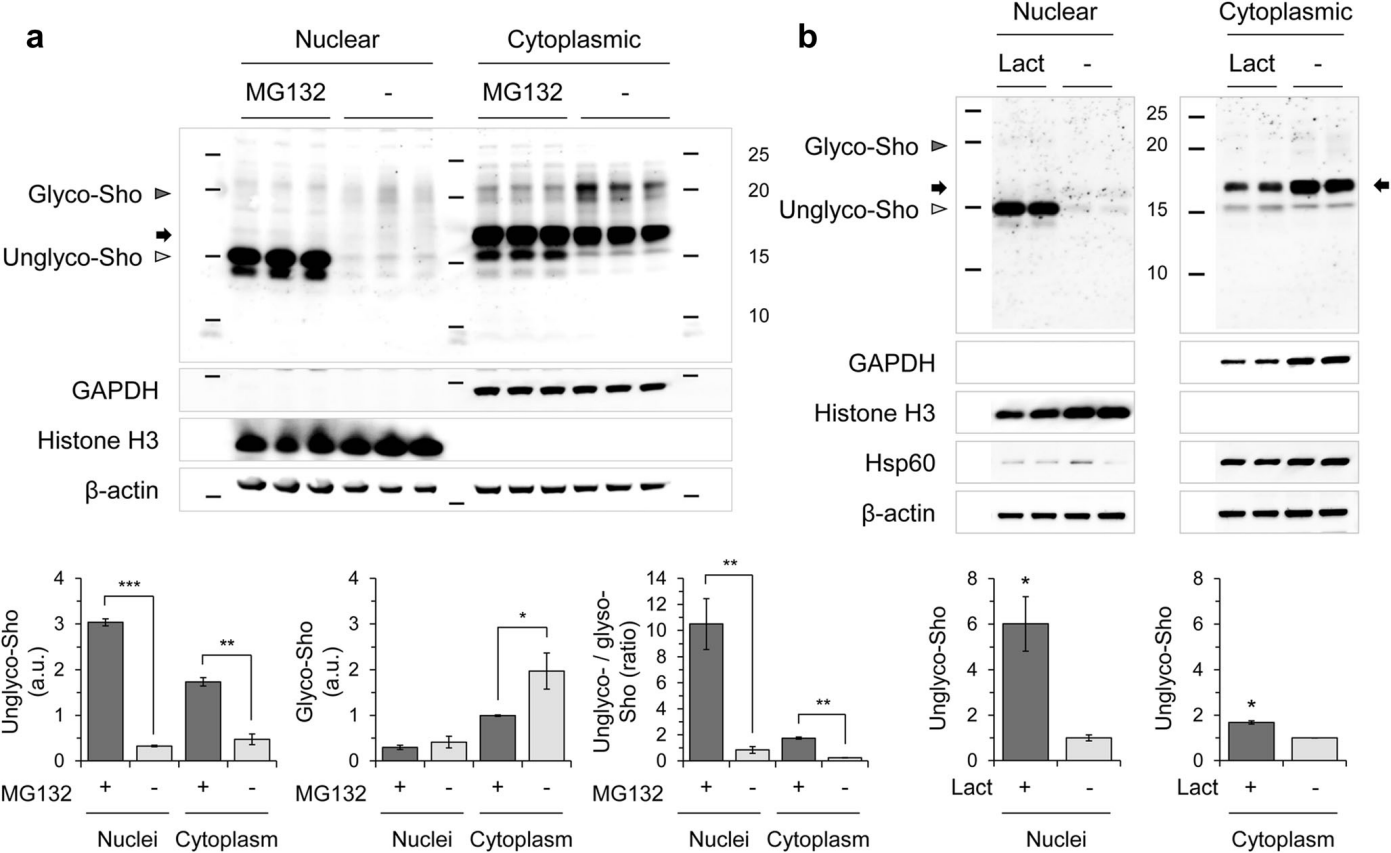
Subcellular fractionation of N2a-*Sprn* treated with MG132 and lactacystin. N2a-*Sprn* cells were treated with **a** MG132 (1 μM) or **b** lactacystin (Lact, 1 μM) for 16 h and harvested after trypsinization (*n* = 3 for MG132, *n* = 2 for Lact). Nuclear and cytoplasmic extracts were prepared by differential detergent fractionation. Nuclear localization of Sho under proteasomal inhibition conditions was analyzed by western blot of the subcellular fractions. Note prominent 15-kDa signal migrating beneath the position of the cross-reactive species. GAPDH, glyceraldehyde 3-phosphate dehydrogenase, a cytoplasmic marker; Histone H3, a nuclear marker; Hsp60, heat shock protein 60, a mitochondrial marker; β-actin, a loading control. Intensity measurement of western blot results was presented. Intensities were normalized to those of histone H3 (nuclear extracts) or GAPDH (cytoplasmic extracts). Error bars represent SD. *p < 0.05, **p < 0.01, and ***p < 0.001 in comparison with vehicle (DMSO) treatment controls. Designations of glycosylated species and a cross-reactive band are as per Fig. 1

The inferred impaired import of Sho into ER under proteasomal inhibition conditions was correlated with notable nuclear translocation of Sho in N2a-*Sprn* cells, similar to that observed in the primary Tg.*Sprn*-MNGCs. The N2a-*Sprn* cells were treated with MG132 and fixed for immunocytochemistry. Sho signals were detected evenly through the cell body under normal culture condition (vehicle controls), whereas nuclear Sho signals were increased by MG132 treatment (Fig. 7a, b). Unlike the situation with Sho translocation, intracellular and cell surface signals of endogenous PrP^C^ were evident in N2a-*Sprn*, regardless of MG132 treatment (Fig. 8a, b). To further verify the nuclear localization of Sho, the nuclear and cytoplasmic fractions of N2a-*Sprn* treated with MG132 or lactacystin were analyzed by western blot. Similar to Tg.*Sprn*-MNGCs (Fig. 5a), glycosylated forms were barely detectable, presumably due to a trypsinization step performed before cell harvesting. The ~ 15-kDa bands, corresponding to the unglycosylated Sho, were detected in the nuclear fractions with both MG132 and lactacystin treatments (Fig. 9a, b). These data indicate that, under condition of proteasomal inhibition, Sho translocates into the nucleus, instead of undergoing post-translational maturation through the secretory pathway.

## Discussion

Earlier work has indicated that general proteostatic pathways may be engaged in response to prion infections of the CNS tissue. Some of these data correspond to decreases in the steady-state levels of Sho and the cellular isoform of prion protein (PrP^C^) [11, 17–20, 45], while others have studied interactions of infectivity-associated isoforms of prion protein (PrP^Sc^) with the proteasome [46, 47] or performed genome-wide analyses to implicate the ubiquitin ligase Hect2D [48]. PrP^Sc^ levels in both central and peripheral neuronal cells were reduced by intracellular proteolytic activities; thus, autophagy stimulation with mTOR inhibitor [49] or ubiquitin-proteasome system (UPS) stimulation with a selective small-molecule inhibitor (IU1) of ubiquitin-specific protease 14 (USP14) [50] decreased accumulation and/or release of PrP^Sc^.

**Fig. 10:**
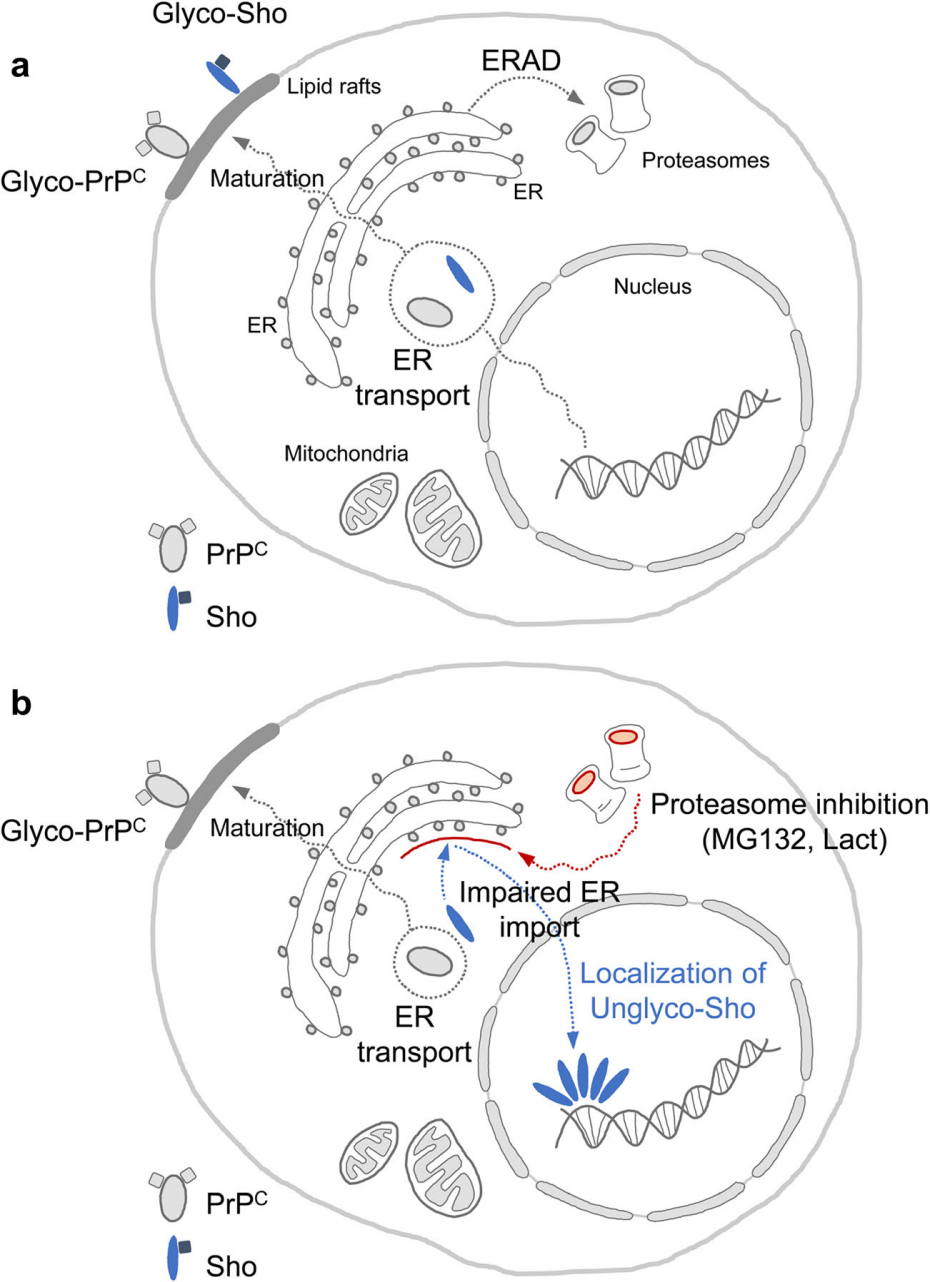
Graphic summary of proteasomal inhibition-induced nuclear localization of Sho. **a** Sho, a highly conserved member of prion protein family, and PrP^C^ are imported into the ER and post-translationally modified with N-linked glycans. The matured glycoproteins are targeted to the lipid rafts of plasma membrane via GPI anchor. Unprocessed or misfolded proteins are eliminated through ER-associated degradation (ERAD) as an ER quality control (ERQC) system. **b** An interference of the ubiquitin-proteasome system (UPS) with proteasome inhibitors, MG132 and lactacystin (Lact), causes an impaired ER import of Sho, however not PrP^C^. Instead of a canonical modification through ER, Sho is localized to the nucleus. Proteins and sugars are indicated by oval and square shapes, respectively

Because of divergent results from the use of inhibitors to assess Sho biogenesis, even in cell culture paradigms, we performed a new series of studies. As noted previously [51], wild-type Sho has few immunogenic epitopes and consequently, it can be difficult to detect, leading to recourse to use of epitope tags and/or overexpression to track its fate. Here, we have employed (a) a neuronal (*Prnp*) promoter transgene (Tg.*Sprn*24551) producing a net level of 2.5x expression (versus 1.0 expression in wild-type mice [19]) and (b) a housekeeping gene (EF-1α) promoter to direct expression of mRNAs encoding untagged wild-type mouse Sho. With these experimental parameters and within different cellular systems, we found that inhibition of the proteasome (with either lactacystin or MG132) invokes a repartitioning effect, rather than a change in the net levels of all Sho species. This effect involves an altered targeting decision for Sho, leading to more substantial levels in the nucleus (summarized in Fig. 10). This stands in contrast to the proposition that glycosylation is a causal determinant of changes in Sho populations [21].

Related to the configuration of experimental paradigms, we also observed massive increases in the levels of all forms of Sho (i.e., glycosylated, unglycosylated, and low molecular weight fragments) in the presence of 3-MA or MG132 when using a human cytomegalovirus (CMV) promoter in uninfected N2a cells or in chronically infected N2a cells (ScN2a) (Fig. S1). Superficially, these data suggest profound regulation of Sho under basal conditions by both proteasomal and autophagic pathways but these data also overlap a prior finding where increases in PrP^C^ levels were achieved by treatments with proteasomal inhibitors when the protein is expressed from the CMV promoter [52]. In line with this work from other labs, the robust across-the-board effect obtained for Sho using CMV promoters was considered an artifact and not pursued further.

Results presented here echo the general concept that Sho has flexibility in terms of its cellular destination [16, 26, 45], sometimes referred to as “dual targeting.” But, rather than having a constitutively leaky signal peptide that leads to cytoplasmic delivery as per the literature for PrP^C^ [22–25], a reduction of Sho expression at the cell surface and an increase of expression in the nucleus were noted following proteasomal inhibition. Also, the details of our findings diverge from some other studies. While repartitioning of Sho to mitochondria is reported for conditions which reduce ER import (as produced by drugs or siRNAs that target the Sec61-containing translocon complex) [26], movement to this destination was not a prominent effect in our studies; cross-contamination of mitochondria into nuclear or cytoplasmic subcellular fractions was assessed by use of anti–heat shock protein 60 (Hsp60) antibody with a presence in cytoplasmic fractions being more notable (Figs. 5a and 9b). However, it remains possible that a subset of Sho lies within mitochondria below our level of detection.

In terms of mechanism, our data do not point to a relocalization whereby mature Sho at the cell surface is redirected internally for nuclear importation. This type of effect involving glycosylated molecules would likely contravene rules for such importation [53, 54]. Instead, we infer repartitioning as a result of nascent protein molecules on ribosomes engaging/not engaging the ER translocon machinery (an idea partly related to other studies that emphasize mitochondrial import [16, 26]). For Sho synthesized on cytoplasmic ribosomes, excision of N- and C-terminal signal peptides will not occur because the relevant endopeptidases are expressed in the secretory pathway. Indeed, the predominant form of Sho detected in MG132-treated cells has a gel mobility slower than two forms of in vitro deglycosylated Sho from untreated cells, indicating that both signal peptides are retained (Fig. 3c, d). This finding is perhaps in accordance with the reciprocal idea that absence of the GPI signal sequence is required for the mitochondrial importation of Sho [26]. Analyses of control cells lacking MG132 treatment demonstrated two approximately equimolar species after PNGase F treatment (“ii” and “iii” in Fig. 3c and d); while the faster-migrating species must correspond to removal of the C-terminal GPI signal peptide, the slower-migrating signal could reflect retention of this signal peptide and hence an inefficiency in processing in MNGCs. Interestingly, cleaved fragments of Sho were observed in neuronal and glial mixed cultures (Tg.*Sprn*-MNGCs) with MG132 or lactacystin treatment (Fig. 5a), but not in N2a-*Sprn* cells (Fig. 9a, b). This data may indicate that, as one of the most active roles of glial cells in the nervous system is phagocytic clearance, glial cells including astrocytes and microglia in MNGCs take up material from granule neurons under conditions of proteasomal inhibition, unlike the situation in the N2a cell monocultures. Examining the glycosylation status of the immunoreactive material in glia and the presence of N1/C1 or N2/C2 fragmentation [55] may be instructive in determining its mechanistic origin from the cytoplasmic or secretory pathway of neurons.

Sho’s N-terminal half is arginine-rich and includes RGG motifs (and degenerate versions thereof) that are predicted to mediate binding to DNA or RNA [28] and work on recombinant Sho fragments revealed that this effect does manifest in vitro [29]. While cell surface full-length Sho can be considered as a potential pattern recognition receptor [56] that could bind to extrinsic RNAs, we found that innate immune ligands had negligible effects upon Sho levels or glycosylation profile (Fig. 2b). On the other hand, when Sho is synthesized from cytoplasmic ribosomes, a latent capacity for nucleic acid binding might come to the fore. In studies using Sho-YFP fusion proteins under resting cell conditions, nuclear location and a nuclear localization signal (NLS) were inferred for Sho [27]. Though these observations align, the biological properties of Sho species when they reach the nucleus are, as yet, unclear. There is now much interest in the low complexity domains of RNA binding proteins and their ability to undergo phase transitions when they aggregate, making transient organelles that are not delimited by lipid bilayers. Since recombinant Sho is natively disordered [11, 14, 26, 27] and has low sequence complexity (Arg, Gly, Ala, and Val residues account for 90% of residues in mouse Sho 25-87), probing for these types of structural transitions might provide a firmer grip on function [57].

In spite of PrP^C^ having its own literature on nucleic acid binding and two putative NLS sequences [58–61], when we examined endogenous PrP^C^ molecules in our experimental paradigms, we did not detect a propensity to alter location or to manifest with different glycotypes (the latter in accord with a prior literature [62, 63]). A simple way to view these findings is in terms of a predominating role for RGG motifs in specifying nucleic acid binding and nuclear localization [27, 29] and the presence (Sho) or absence (PrP^C^) of these motifs prior to the central hydrophobic region of the respective proteins. Also, with full-length forms of Sho being less than 20kDa, they are considerably below a cutoff of 30–60–kDa for passive diffusion into nucleus [64]. The notion of divergence in function following from different propensity to relocalize under conditions of compromised proteasomal activity can be reconciled with results of a genetic interrogation, namely the viability of *Prnp*^0/0^ + *Sprn*^0/0^ double knockout mice [13, 30].

Overarching goals in this area are to define the association between allelic forms of Sho and two varieties of Creutzfeldt-Jakob disease [65], the cause of coordinated changes in Sho and PrP^C^ levels in scrapie disease, and the possible role of general clearance pathways for glycoproteins. An earlier notion derived from studies of scrapie infections is that Sho and PrP^C^ [19, 20] are substrates for the same, albeit unidentified protease system [20]. If this unidentified system were none other than the proteasomal system, then a number of observations on PrP^C^ and PrP^Sc^ and preclinical changes in infected animals might be tied together [20, 45–47, 66]. On the other hand, the divergent observations that prion infection diminishes net levels for Sho (and PrP^C^) [20, 45], whereas proteasomal inhibition used here causes a balanced redistribution, suggest that this unifying logic is premature or an oversimplification. What is perhaps most surprising here is that studies with the intention to probe the proteostatic pathways degrading members of the mammalian PrP superfamily have revealed Sho not merely a passive substrate, but, with the varying location, as an indicator of proteostatic status. Our data point to engagement/non-engagement of the ER translocon machinery as the decision point for alternative targeting, suggesting that natural signal peptide polymorphisms in humans and sheep [65, 67] will impact the endpoints in the assays presented here and provide broader illumination on Sho biology.

## Electronic Supplementary Material

Fig. S1 **Effects of 3-MA and MG132 on Sho expression driven by CMV promoter.** Chronically prion-infected N2a cells (ScN2a) and uninfected N2a cells were transfected with gene expression constructs, in which Sho expression is driven by the human cytomegalovirus (CMV) promoter. (**a**) and (**b**) ScN2a cells transiently transfected with the Sho construct by chemically enhanced electroporation (ScN2a-*Sprn*) were treated with 3-MA at the indicated concentrations. Dose- (**a**) and time-dependent (**b**) effect of 3-MA on Sho expression were determined by western blot. (**c**) and (**d**) N2a cells stably expressing Sho under control of CMV promoter (N2a-*Sprn*) were treated with 3-MA (**c**) or MG132 (**d**) at the indicated concentrations. Sho and PrP^C^ expression were analyzed by western blot. (**e**) ScN2a-*Sprn* were treated with MG132 at the indicated concentrations and Sho expression was determined by western blot. Glycosylated species and a cross-reactive band are designated as per Fig. 1. (PNG 348 kb)

High resolution image (TIF 6311 kb)
