# Supplementary figures and images for: Proteasomal Inhibition Redirects the PrP-Like Shadoo Protein to the Nucleus
Source: Mol Neurobiol. 2019 May 25;56(11):7888–904. doi: 10.1007/s12035-019-1623-1 (PMC6815274; doi:10.1007/s12035-019-1623-1)

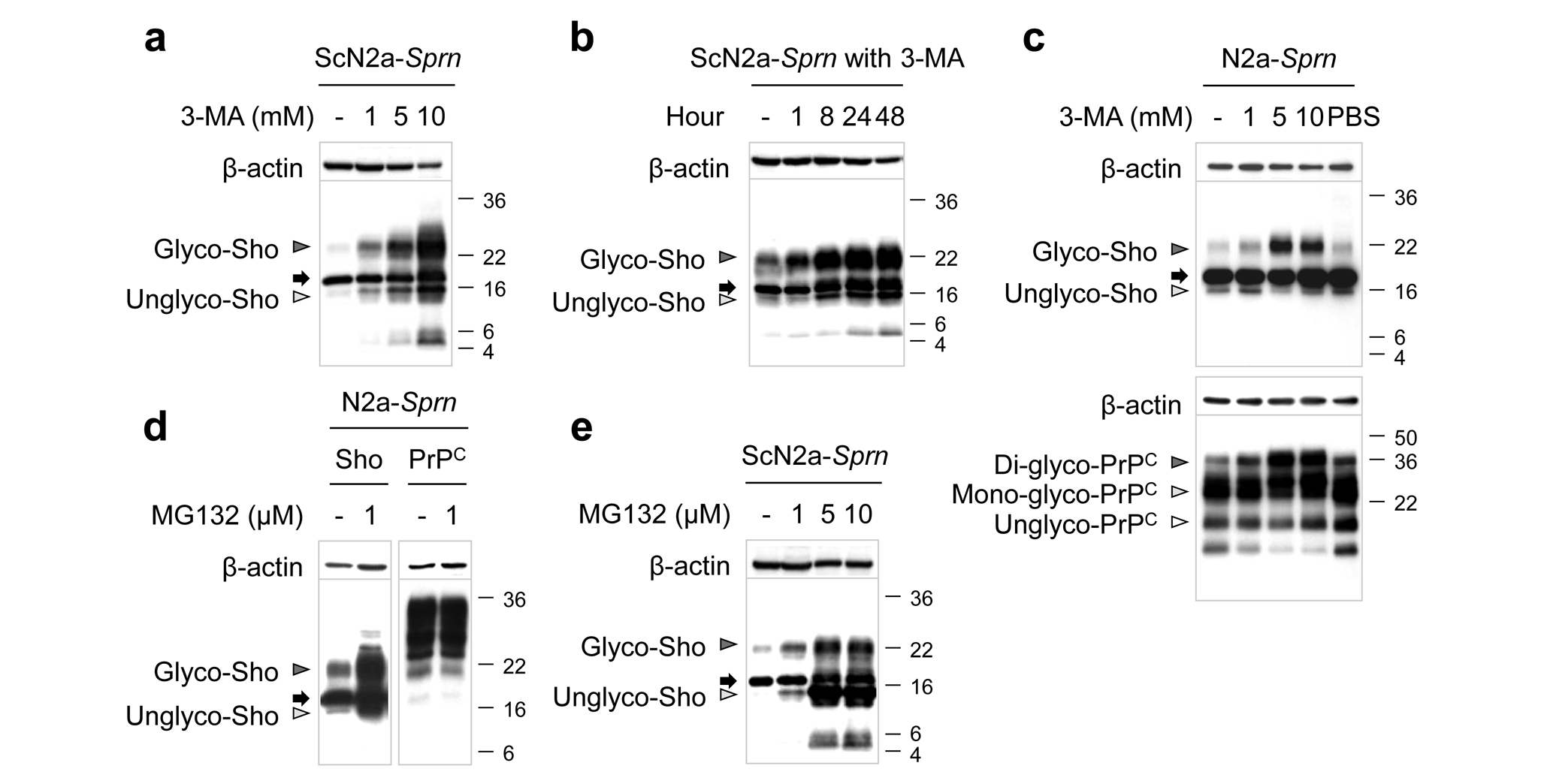

Supplement: Supplementary file 1 — Effects of 3-MA and MG132 on Sho expression driven by CMV promoter. Chronically prion-infected N2a cells (ScN2a) and uninfected N2a cells were transfected with gene expression constructs, in which Sho expression is driven by the human cytomegalovirus (CMV) promoter. (a) and (b) ScN2a cells transiently transfected with the Sho construct by chemically enhanced electroporation (ScN2a-Sprn) were treated with 3-MA at the indicated concentrations. Dose- (a) and time-dependent (b) effect of 3-MA on Sho expression were determined by western blot. (c) and (d) N2a cells stably expressing Sho under control of CMV promoter (N2a-Sprn) were treated with 3-MA (c) or MG132 (d) at the indicated concentrations. Sho and PrPC expression were analyzed by western blot. (e) ScN2a-Sprn were treated with MG132 at the indicated concentrations and Sho expression was determined by western blot. Glycosylated species and a cross-reactive band are designated as per Fig. 1. (PNG 348 kb) [file 12035_2019_1623_Fig11_ESM.png]

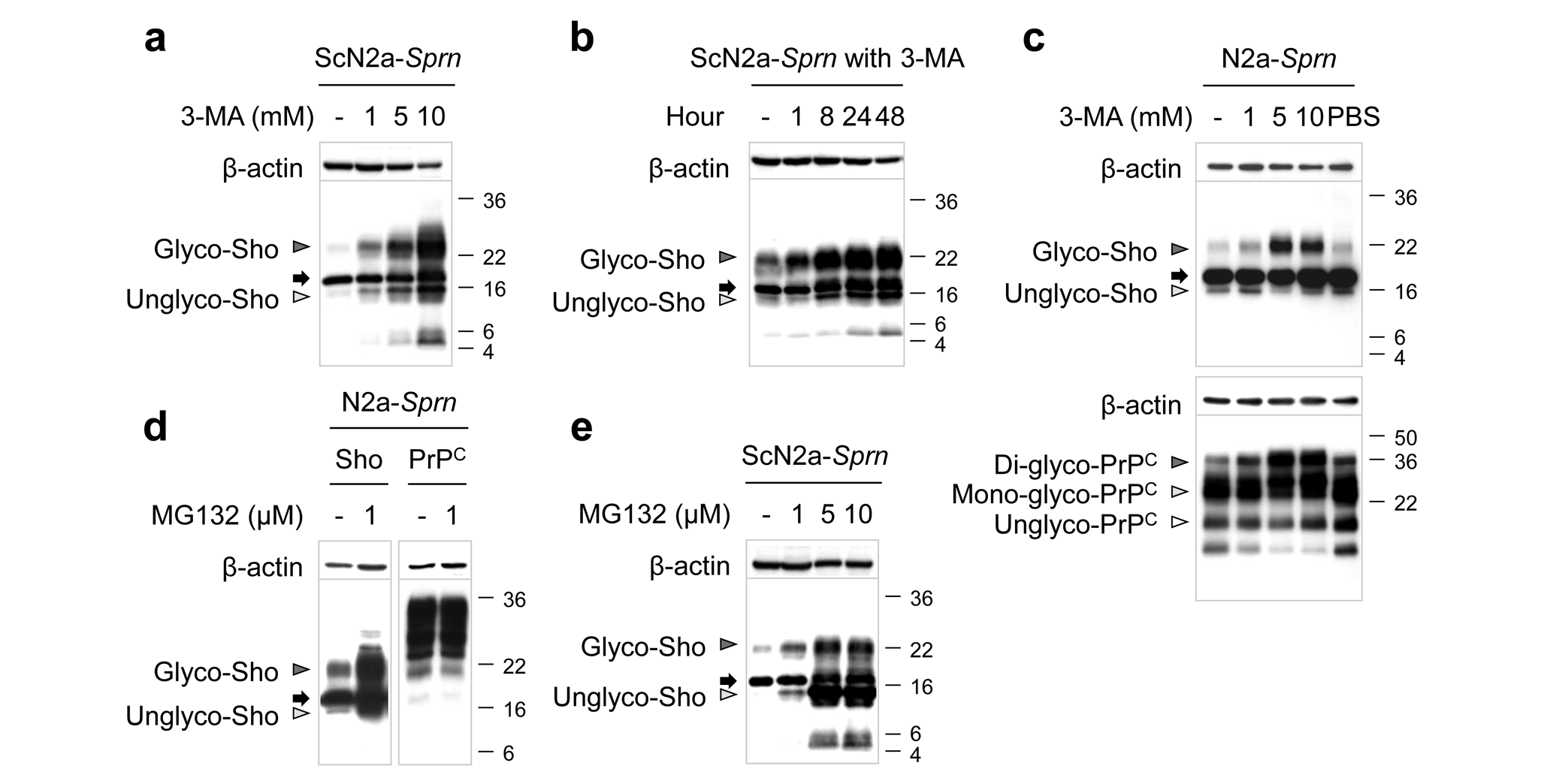

Supplement: Supplementary file 2 — High resolution image (TIF 6311 kb) [file 12035_2019_1623_MOESM1_ESM.tif]
